# TRANSCRIPTOMIC ANALYSIS OF PROGERIA MODEL, KLOTHO KO MOUSE AND THE THERAPEUTIC EFFECT OF MITOCHONIC ACID 5

**DOI:** 10.1093/geroni/igad104.3417

**Published:** 2023-12-21

**Authors:** Yoshiyasu Tongu, Tomoko Kasahara, Takaaki Abe

**Affiliations:** Tohoku University, Sendai, Miyagi, Japan; Tohoku University Graduate School of Medicine, Sendai, Miyagi, Japan; Tohoku University, Sendai, Miyagi, Japan

## Abstract

Klotho is recognized as a protein that enhances longevity, and its deficiency leads to symptoms resembling progeria. The Klotho KO mouse displays several aging-related phenotypes similar to those observed in humans and typically dies by 8-10 weeks old. Klotho is expressed primarily in the kidney and the brain, which leads to severe progeria. We’ve recently developed a novel mitochondria-homing drug called Mitochonic Acid (MA)-5, which can penetrate the blood-brain barrier and is currently in Phase 1 clinical trials. A decline in mitochondrial function is a well-documented characteristic of aging and is considered a major factor in the aging process. To explore the potential benefits of MA-5 on aging-related symptoms, we administered the drug orally to Klotho KO mice from 4 weeks old and studied them until 7 weeks old. We observed that levels of uremic creatinine and BUN were elevated in the Klotho KO mice, but MA-5 treatment brought about significant improvements. Pathological examinations and snRNA-seq data suggest that kidney fibrosis caused by Klotho KO might be behind the renal dysfunction, and this is mitigated by MA-5. Furthermore, the result of snRNA-seq in brains shows the abnormalities occurring in the brains of Klotho KO mice which leads to progeria and the therapeutic effect of MA-5 on them. Given the role of mitochondrial dysfunction in aging symptoms, our drug presents as a promising candidate to treat age-related renal failure, and a range of other aging manifestations.

